# Association of *Egr*3 genetic polymorphisms and coronary artery disease in the Uygur and Han of China

**DOI:** 10.1186/1476-511X-13-84

**Published:** 2014-05-21

**Authors:** Xia Li, Yi-Tong Ma, Xiang Xie, Yi-Ning Yang, Xiang Ma, Ying-Ying Zheng, Shuo Pan, Fen Liu, Bang-Dang Chen

**Affiliations:** 1 Department of Cardiology, First Affiliated Hospital of Xinjiang Medical University, Urumqi, Xinjiang 830054, China; 2 Xinjiang Key Laboratory of Cardiovascular Disease Research, Urumqi, Xinjiang 830054, China

**Keywords:** *Egr*3, Coronary artery disease, Polymorphism

## Abstract

**Background:**

Endothelial cell activation and dysfunction are the foundation of atherosclerosis, including coronary artery disease (CAD). Endothelial cell activation is mediated by the level of gene transcription. Early growth response 3 (*Egr*3) is a critical determinant of vascular endothelial growth factor (VEGF) signalling in activated endothelial cells. If endothelial cells are excessively activated, it may lead to vasculopathic diseases, such as pathologic angiogenesis, inflammation, and atherosclerosis. The aim of the present study was to assess the association between the *Egr*3 gene polymorphisms and CAD.

**Methods:**

Two independent case–control studies that involved the Han group (409 CAD patients and 351 control subjects) and the Uygur group (299 CAD patients and 303 control subjects) analysed the relationship between *Egr3* SNPs (rs1996147 and rs1008949) and CAD. Genotyping was undertaken using the TaqMan SNP genotyping assay.

**Results:**

The entire Uygur group and the males in the Uygur group showed a higher frequency of the A allele (rs1996147) in CAD patients than in the control subjects (*P* = 0.003 and *P* = 0.005, respectively). Additionally, the distribution of the recessive model of rs1996147 (AA vs GG + AG) for the total sample and the males was significantly different between CAD patients and control participants (*P* = 0.002 and *P* = 0.003, respectively), and the difference remained statistically significant following multivariate adjustment (Total: OR = 1.705; 95% CI: 1.166-2.494, *P* = 0.006; males: OR = 1.908, 95% CI: 1.189-3.062, *P* = 0.007). However, for Uygur females, we did not observe a difference in the allele frequency or genotypic distribution of rs1996147 between CAD patients and control participants. Similarly, the distribution of the rs1996147 allele frequency or genotypes showed no significant difference between patients with CAD and control participants in the Han group. The distribution of rs1008949 genotypes, dominant model, recessive model, and allele frequency did not show a significant difference between patients with CAD and the control subjects in the Han and Uygur groups.

**Conclusion:**

rs1996147 may be a novel polymorphism of the *Egr*3 gene associated with CAD in males of the Chinese Uygur population.

## Background

Coronary artery disease (CAD) is thought to be a complex multifactorial and polygenic disorder resulting from interactions between an individual’s genetic makeup and various environmental factors [1]. Epidemiology investigations have revealed that some genetic variants, such as polymorphisms in the renin angiotensin system [2], apolipoprotein E [3], blood coagulation factors [4], and inflammation factors [5], increase the risk of coronary artery disease (CAD).

The early growth response (*Egr*) family of transcription factors (*Egr*1 to *Egr*4) is the nuclear regulator of endothelial cell activation. *Egr*1, 2, and 3 are transcriptional activators, whereas *Egr*4 is a transcriptional repressor. The activation of target gene transcription by *Egr* family members requires their de novo protein synthesis [6]. *Egr*3 is a member of a zinc finger transcription factor subfamily, which was found to be strongly upregulated by vascular endothelial growth factor (VEGF) in an oligonucleotide microarray screen of endothelial cells [7]. Recent evidence points to a role for *Egr*3 in transducing signals in endothelial cells and is one of the most highly inducible genes in VEGF–treated endothelial cells [8,9]. The role of VEGF in atherosclerosis is a subject of debate in the literature. Some studies show that the administration of recombinant human VEGF in animals enhances the progression of atherosclerotic plaque [10], whereas others observed that it acts as an anti-atherosclerotic factor, promoting re-endothelisation, reducing intimal thickening and preventing thrombus formation [11]. Suehiro and colleagues [6] show that VEGF-induced endothelial activities are mediated by the activation of the *Egr*3 transcription factor. *Egr*3 is rapidly induced by extracellular stimuli and has been implicated in the proliferation and differentiation of several different cell types, including endothelial cells [6]. Liu D et al. [7] reported that VEGF induced a rapid increase in *Egr*-dependent transcriptional activation mediated via its major signalling receptor, tyrosine-kinase receptors KDR (VEGFR2), and the protein kinase C (PKC) pathway. The inhibition of *Egr*3 gene expression by RNA interference was effective in inhibiting basal and VEGF-induced *Egr*3 gene expression, and it also inhibited VEGF-mediated endothelial cell proliferation, migration, and tubulogenesis. These findings indicate that *Egr*3 has an essential downstream role in VEGF-mediated endothelial functions that lead to angiogenesis and may have particular relevance for adult angiogenic processes involved in vascular repair and neovascular disease. However, the role of *Egr*3 in atherosclerosis remains unknown.

Previous studies have shown that the quantification of amplified *Egr*3 fragments leads to a significant increase in expression following 3 h of ischemia, with a maximum increase (5-fold) at 24 h in a mouse survival model of ischemia [12]. Therefore, *Egr*3 may be an important contributor to the development and progression of atherosclerotic disease. Accordingly, we screened for possible mutations and polymorphisms of the *Egr*3 gene and assessed the association between the genotypes of this gene and CAD in a Chinese Uygur and Han population.

## Methods

All patients gave written informed consent and explicitly provided permission for DNA analyses as well as for the collection of relevant clinical data. The study protocols were approved by the Ethics Committees of the First Affiliated Hospital of Xinjiang Medical University.

### Subjects

Two patient populations (Han and Uygur) with CAD were studied independently. All patients were recruited at the First Affiliated Hospital of Xinjiang Medical University from 2007 to 2010. The study participants included 409 Han patients and 299 Uygur patients diagnosed with CAD. All of the CAD patients were defined by angiographic means (main coronary artery stenosis of >50%). For each CAD patient group, we selected healthy controls matched for ethnicity, sex, and age. Control subjects were selected from the cardiovascular risk survey (CRS) [13,14]. Briefly, the CRS is a prospective, observational cohort study designed to investigate the prevalence, incidence, and risk factors of cardiovascular disease in the Han, Uygur, and Kazakh populations in Xinjiang of China from October 2007 to March 2010. Control participants included Han (n = 351) and Uygur (n = 303) individuals who did not have coronary vessel stenosis and did not show clinical or electrocardiographic evidence of myocardial infarction (MI) or CAD. All patients with impaired renal function, malignancy, connective tissue disease, schizophrenia, or chronic inflammatory disease were excluded. Diabetes, hypertension, hyperlipidemia, smoking, and alcohol consumption were defined as previously described [15-17].

### Biochemical analysis

We measured the plasma concentration of blood triglycerides (TG), total cholesterol (TC), high-density lipoprotein (HDL), and low-density lipoprotein (LDL) using standard methods in the Clinical Laboratory Department of the First Affiliated Hospital, Xinjiang Medical University as described previously [17,18].

### SNP selection

We selected two single-nucleotide polymorphisms (SNPs) in the human *Egr*3 gene as markers for assessment of genetic association. There are 103 SNPs in the human *Egr3* gene listed in the National Center for Biotechnology Information SNP database (http://www.ncbi.nlm.nih.gov/SNP). Using the Haploview 4.2 software and the HapMap phrase II database, we obtained two tagging SNPs (rs1996147, rs1008949) for Chinese Hans and Uygurs using minor allele frequency (MAF) ≥0.05 and linkage disequilibrium patterns, with r^2^ ≥ 0.8 as a cut-off.

### Genotyping

Blood samples were collected from all participants. Genomic DNA was extracted from peripheral blood leukocytes using phenol and chloroform [19]. Genotyping was undertaken using TaqMan SNP genotyping assays (Applied Biosystems [ABI], Foster City, California), which were performed using Taq amplification [20]. In the first step of the 5’ nuclease assay, allele-specific fluorogenic probes were hybridised to the template. Subsequently, the 5’ nuclease activity of the Taq polymerase made it possible for discrimination during the polymerase chain reaction (PCR). The probes contain a 3’minor groove-binding group that hybridises to single-stranded targets and has greater sequence specificity than the standard DNA probes. This reduces nonspecific probe hybridisation, thereby resulting in low background fluorescence for the 5’ nuclease PCR assay (TaqMan; Applied Biosystems). Cleavage results in the increased emission of a reporter dye. Each 5’ nuclease assay requires two unlabelled PCR primers and two allele-specific probes. Each probe is labelled with two reporter dyes at the 5’ end. In the present study, VIC and FAM were used as the reporter dyes. The primers and probes used in the TaqMan SNP genotyping assays (Applied Biosystems) were chosen based on information from the ABI website (http://appliedbiosystems.com.cn/).

PCR amplification was performed using 6 μL of TaqMan Universal Master Mix, No AmpErase UNG (2×; ABI) in a 12-μL final reaction volume containing 2 ng of DNA, 0.22 μL of TaqMan SNP genotyping assay mix (20× or 40×), primers at a concentration of 900 nmol/L each, and probes at a final concentration of 200 nmol/L each. The thermal cycling conditions were as follows: 50°C for 2 minutes, 95°C for 10 minutes, 40 cycles of 95°C for 15 seconds, and 62°C for 1 minute. Thermal cycling was performed using the GeneAmp 7900 system. Plates were read on the sequence detection systems (SDS) automation controller software v2.4 (ABI).

### Statistical analysis

Data analysis was performed using Statistical Package for Social Sciences SPSS for Windows (version 17.0). Hardy-Weinberg equilibrium was assessed using *X*^2^ analysis. All continuous variables were expressed as the mean ± standard deviation (SD). The differences between the CAD patients and the control participants were assessed using independent samples *t* test. Differences in enumeration data between CAD patients and control participants were analysed using the X^2^ test. Categorical variables, such as allele and genotype frequencies among CAD cases and controls were compared using the chi-square test. Additionally, logistic regression analysis was performed to assess the odds ratio (OR) and its 95% confidence interval (CI) as a measure of the association between the *Egr3* polymorphism and risk of CAD. A value of *P* < 0.05 was considered significant.

## Results

Table 1 shows the clinical characteristics of CAD patients and control subjects in the Han population. For the total sample and for male and female participants, there were no significant differences in age, body mass index (BMI), pulse and serum concentration of TG and HDL-C between patients with CAD and control participants. For total subjects, males, and females, the following values were significantly higher for the CAD patients compared with the control subjects: LDL-C, incidence of diabetes, and smoking (all *P* < 0.05). For total subjects and males, the serum concentration of total cholesterol was significantly higher for the CAD patients compared with the control subjects. However, for females, there was no difference between CAD patients and control participants. For males, the prevalence of essential hypertension (EH) was not significant (*P* > 0.05); however, it was higher for patients with CAD than for the control participants, total subjects and females (*P* <0.05).

**Table 1:** Characteristics of study participants in the Han population

	**Total**			**Men**			**Women**		
	**CAD patients**	**Control subjects**	***P *** **value**	**CAD patients**	**Control subjects**	***P *** **value**	**CAD patients**	**Control subjects**	***P *** **value**
Number of subjects(n)	409	351		300	179		109	172	
Age(years), mean(SD)	57.06(10.41)	56.58(9.5)	0.510	56.97(10.89)	56.03(10.05)	0.348	57.64(9.75)	57.16(9.35)	0.680
BMI (kg/m^2^),mean(SD)	25.60(3.29)	26.06(3.51)	0.060	25.72(3.34)	26.47(3.33)	0.016	25.26(3.13)	25.63(3.66)	0.380
Pulse (beats/min),mean(SD)	72.51(10.90)	72.97(10.28)	0.547	72.19(10.94)	74.09(11.46)	0.072	73.34(10.79)	71.81(8.77)	0.185
Total cholesterol (mmol/L),mean(SD)	4.50(0.91)	4.37(0.99)	0.047*	4.44(0.88)	4.25(1.25)	0.031*	4.68(0.97)	4.46(0.93)	0.058
Triglycerides (mmol/L),mean(SD)	1.84(1.14)	1.77(1.25)	0.483	1.80(1.08)	1.86(1.45)	0.058	1.92(1.30)	1.68(0.99)	0.085
LDL (mmol/L),mean(SD)	2.49(0.91)	2.13(0.83)	<0.001*	2.53(0.77)	2.32(0.87)	0.006*	2.47(1.02)	2.03(0.78)	<0.001*
HDL (mmol/L),mean(SD)	1.17(0.49)	1.23(0.51)	0.095	1.13(0.51)	1.15(0.54)	0.733	1.26(0.39)	1.31(0.47)	0.374
EH,n (%)	220(53.8)	163(46.4)	0.043*	151(50.3)	80(44.7)	0.232	69(63.3)	83(48.3)	0.014*
DM (%)	102(24.9)	39(11.1)	<0.001*	70(23.3)	20(11.2)	<0.001*	32(29.4)	19(11.0)	<0.001*
Smoking (%)	230(56.2)	87(24.8)	<0.001*	208(69.3)	83(46.4)	<0.001*	22(20.2)	4(2.3)	<0.001*

Continuous variables are expressed as mean ± s.d. Categorical variables are expressed as percentages.

CAD, coronary artery disease; BMI, body mass index; LDL, Low-density lipoprotein; HDL, high-density lipoprotein; EH, essential hypertension; DM, diabetes mellitus.

The *P* value of the continuous variables was calculated by the independent samples *t* test.

The *P* value of the categorical variables was calculated by X^2^ test. *P < 0.05.

Table 2 shows the clinical characteristics of CAD patients and control subjects in the Uygur population. For total subjects, males, and females, the following values were significantly higher for the CAD patients compared with the control subjects: the serum concentration of total cholesterol and LDL-C and smoking (*P* < 0.05). For total subjects and male and female participants, there was no significant difference in the following variables between CAD patients and control subjects: age, BMI, pulse, triglycerides, EH, and DM (all *P* > 0.05). The serum concentration of HDL-C only showed a significant difference between patients with CAD and female control participants in the Uygur population (*P* < 0.05).

**Table 2:** Characteristics of study participants in the Uygur population

	**Total**			**Men**			**Women**		
	**CAD patients**	**Control subjects**	***P *** **value**	**CAD patients**	**Control subjects**	***P *** **value**	**CAD patients**	**Control subjects**	***P *** **value**
Number of subjects(n)	299	303		198	196		101	107	
Age (years),mean(SD)	57.32(8.54)	56.78(8.89)	0.447	56.82(8.49)	56.02(8.56)	0.352	58.02(8.62)	57.13(8.96)	0.467
BMI (kg/m^2^),mean(SD)	26.05(3.55)	25.89(4.21)	0.615	26.29(3.59)	26.11(4.06)	0.641	25.95(3.02)	25.02(4.31)	0.075
Pulse (beats/min),mean(SD)	76.06(10.49)	75.02(11.93)	0.212	75.70(10.51)	74.74(12.80)	0.420	77.08(10.43)	75.52(10.20)	0.278
Total cholesterol (mmol/L),mean(SD)	4.74(0.94)	4.25(0.90)	<0.001*	4.72(0.83)	4.13(0.90)	<0.001*	4.77(1.23)	4.45(0.90)	0.023*
Triglycerides (mmol/L),mean(SD)	1.85(0.95)	1.82(1.12)	0.766	1.78(0.93)	1.84(1.08)	0.578	1.98(0.98)	1.80(1.21)	0.233
LDL (mmol/L),mean(SD)	2.74(0.81)	2.48(1.12)	0.001*	2.71(0.79)	2.45(1.26)	0.017*	2.80(0.84)	2.53(0.80)	0.019*
HDL (mmol/L),mean(SD)	0.99(0.33)	1.06(0.53)	0.054	0.99(0.33)	1.05(0.63)	0.232	1.01(0.32)	1.09(0.27)	0.039*
Hypertension (%)	134(44.8)	123(40.6)	0.295	86(43.4)	77(39.5)	0.427	48(47.5)	46(43.0)	0.511
DM (%)	58(19.4)	41(13.5)	0.052	38(19.2)	25(12.8)	0.081	20(19.8)	16(15.0)	0.356
Smoking (%)	195(65.2)	116(38.6)	<0.001*	145(73.2)	96(49.0)	<0.001*	50(49.5)	21(19.6)	<0.001*

Continuous variables are expressed as mean ± s.d. Categorical variables are expressed as percentages. CAD, coronary artery disease; BMI, body mass index; LDL, Low-density lipoprotein; HDL, high-density lipoprotein; DM, diabetes mellitus; The *P* value of the continuous variables was calculated by the independent samples *t* test. The *P* value of the categorical variables was calculated by X^2^ test. *P < 0.05.

Tables 3 and 4 show the distribution of the genotypes and alleles for the two SNPs of *Egr*3 gene in the Han and Uygur populations. The genotypic distribution for each of the SNPs was in agreement with the predicted Hardy–Weinberg equilibrium values for both ethnicities (*P* > 0.05 in the CAD and control groups, data not shown).

**Table 3:** Genotyping and allele distributions in control subjects and patients with CAD in the Han population

	**Total**			**Men**			**Women**		
**Variants**	**CAD patients**	**Control subjects**	***P *** **value**	**CAD patients**	**Control subjects**	***P *** **value**	**CAD patients**	**Control subjects**	***P *** **value**
**rs1996147**									
Genotyping									
AA	45(11.0%)	49(14.0%)		35(11.7%)	28(15.6%)		10(9.2%)	21(12.2%)	
AG	199(48.7%)	156(44.4%)		150(50.0%)	77(43.1%)		49(45.0%)	79(45.9%)	
GG	165(40.3%)	146(41.6%)	0.346	115(38.3%)	74(41.3%)	0.252	50(45.8%)	72(41.9%)	0.664
Dominant model									
GG	165(40.3%)	146(41.6%)		115(38.3%)	74(41.3%)		50(45.8%)	72(41.9%)	
AA + AG	244(59.7%)	205(58.4%)	0.726	185(61.7%)	105(58.7%)	0.515	59(54.2%)	100(58.1%)	0.509
Recessive model									
AA	45(11.0%)	49(14.0%)		35(11.7%)	28(15.6%)		10(9.2%)	21(12.2%)	
GG + AG	364(89.0%)	302(86.0%)	0.217	265(88.3%)	151(84.4%)	0.213	99(90.8%)	151(87.8%)	0.429
Additive model									
AG	199(48.7%)	156(44.4%)		150(50.0%)	77(43.1%)		49(45.0%)	79(45.9%)	
AA + GG	210(51.3%)	195(55.6%)	0.246	150(50.0%)	102(56.9%)	0.139	60(55.0%)	93(54.1%)	0.873
Allele									
A	289(35.3%)	254(36.2%)		220(36.7%)	133(37.2%)		69(31.7%)	121(35.2%)	
G	529(64.7%)	448(63.8%)	0.730	380(63.3%)	225(62.8%)	0.881	149(68.3%)	223(64.8%)	0.390
**rs1008949**									
Genotyping									
CC	112(27.4%)	99(28.2%)		85(28.3%)	55(30.7%)		30(27.5%)	44(25.6%)	
CT	204(49.9%)	173(49.3%)		149(49.7%)	84(46.9%)		55(50.5%)	89(51.7%)	
TT	93(22.7%)	79(22.5%)	0.969	69(23.0%)	40(22.4%)	0.817	24(22.0%)	39(22.7%)	1.000
Dominant model									
CC	112(27.4%)	99(28.2%)		85(28.3%)	55(30.7%)		30(27.5%)	44(25.6%)	
TT + CT	297(72.6%)	252(71.8%)	0.801	218(72.7%)	124(69.3%)	0.532	79(72.5%)	128(74.4%)	0.719
Recessive model									
TT	93(22.7%)	79(22.5%)		69(23.0%)	40(22.4%)		24(22.0%)	39(22.7%)	
CC + CT	316(77.3%)	272(77.5%)	0.939	234(77.0%)	139(77.6%)	0.914	85(78.0%)	133(77.3%)	0.995
Additive model									
CT	204(49.9%)	173(49.3%)		149(46.7%)	84(46.9%)		55(50.5%)	89(51.7%)	
CC + TT	205(50.1%)	178(50.7%)	0.871	154(51.3%)	95(53.1%)	0.633	54(49.5%)	83(48.3%)	0.982
Allele									
C	428(52.3%)	371(52.8%)		319(53.2%)	194(54.2%)		115(52.8%)	177(51.5%)	
T	390(47.7%)	331(47.2%)	0.838	287(47.8%)	164(45.8%)	0.641	103(47.2%)	167(48.5%)	0.993

CAD, coronary artery disease; SNP, single-nucleotide polymorphism.

**Table 4:** Genotyping and allele distributions in control subjects and patients with CAD in the Uygur population

	**Total**			**Men**			**Women**		
**Variants**	**CAD patients**	**Control subjects**	***P *** **value**	**CAD patients**	**Control subjects**	***P *** **value**	**CAD patients**	**Control subjects**	***P *** **value**
**rs1996147**									
Genotyping									
AA	109(36.5%)	75(24.7%)		77(38.9%)	49(25.0%)		32(31.7%)	26(24.3%)	
AG	142(47.5%)	156(51.5%)		90(45.5%)	98(50.0%)		52(51.5%)	58(54.2%)	
GG	48(16.0%)	72(23.8%)	0.003*	31(15.6%)	49(25.0%)	0.005*	17(16.8%)	23(21.5%)	0.433
Dominant model									
GG	48(16.0%)	72(23.8%)		31(15.6%)	49(25.0%)		17(16.8%)	23(21.5%)	
AA + AG	251(84.0%)	231(76.2%)	0.018*	167(84.4%)	147(75.0%)	0.021*	84(83.2%)	84(78.5%)	0.394
Recessive model									
AA	109(36.5%)	75(24.7%)		77(38.9%)	49(25.0%)		32(31.7%)	26(24.3%)	
GG + AG	190(63.5%)	228(75.3%)	0.002*	121(61.1%)	147(75.0%)	0.003*	69(68.3%)	81(75.7%)	0.235
Additive model									
AG	142(47.5%)	156(51.5%)		90(45.5%)	98(50.0%)		52(51.5%)	58(54.2%)	
AA + GG	157(52.5%)	147(48.5%)	0.327	108(54.5%)	98(50.0%)	0.366	49(48.5%)	49(45.8%)	0.694
Allele									
A	360(60.2%)	306(50.5%)		244(61.6%)	196(50.0%)		116(57.4%)	110(51.4%)	
G	238(39.8%)	300(49.5%)	0.001*	152(38.4%)	196(50.0%)	0.001*	86(42.6%)	104(48.6%)	0.218
**rs1008949**									
Genotyping									
CC	118(39.5%)	104(34.3%)		77(38.9%)	66(33.7%)		41(40.6%)	38(35.5%)	
CT	136(45.5%)	152(50.2%)		95(48.0%)	98(50.0%)		41(40.6%)	54(50.5%)	
TT	45(15.0%)	47(15.5%)	0.409	26(13.1%)	32(16.3%)	0.472	19(18.8%)	15(14.0%)	0.334
Dominant model									
CC	118(39.5%)	104(34.3%)		77(38.9%)	66(33.7%)		41(40.6%)	38(35.5%)	
TT + CT	181(60.5%)	199(65.7%)	0.191	121(61.1%)	130(66.3%)	0.282	60(59.4%)	69(64.5%)	0.451
Recessive model									
TT	45(15.0%)	47(15.5%)		26(13.1%)	32(16.3%)		19(18.8%)	15(14.0%)	
CC + CT	254(85.0%)	256(84.5%)	0.875	172(86.9%)	164(83.7%)	0.371	82(81.2%)	92(86.0%)	0.350
Additive model									
CT	136(45.5%)	152(50.2%)		95(48.0%)	98(50.0%)		41(40.6%)	54(50.5%)	
CC + TT	163(54.5%)	151(49.8%)	0.267	103(52.0%)	98(50.0%)	0.688	60(59.4%)	53(49.5%)	0.153
Allele									
C	372(62.2%)	360(59.4%)		249(62.9%)	230(58.7%)		123(60.9%)	130(60.7%)	
T	226(37.8%)	246(40.6%)	0.319	147(37.1%)	162(41.3%)	0.227	79(39.1%)	84(39.3%)	0.976

CAD, coronary artery disease; SNP, single-nucleotide polymorphism; *P < 0.05.

For the total participants in the Uygur group, the distribution of rs1996147 genotypes showed a significant difference between patients with CAD and control participants (*P* = 0.003). Additionally, the distribution of the dominant model (GG vs AA + AG), recessive model (AA vs GG + AG), and allele frequency of rs1996147 showed significant differences between patients with CAD and control subjects (P = 0.018, P = 0.002, and P = 0.001, respectively). For the males in the Uygur group, the distribution of rs1996147 genotypes, dominant model (GG vs AA + AG), recessive model (AA vs GG + AG), and allele frequency of rs1996147 were significantly higher in the CAD group than in the control participants (*P* = 0.005, *P* = 0.021, *P* = 0.003, and *P* = 0.001, respectively). There was no significant difference between the CAD group and the control subjects in the Uygur females in the distribution of rs1996147 genotypes, dominant model (GG vs AA + AG), recessive model (AA vs GG + AG), and allelic distribution (*P* > 0.05). Moreover, for total participants, males and females in the Uygur group, the distribution of rs1008949 genotypes, dominant model (CC vs TT + CT), recessive model (TT vs AA + CT), and allele frequency did not show significant differences between patients with CAD and control subjects (*P* > 0.05). Similarly, for total participants, males and females in the Han group, the distribution of two SNPs (rs1996147 and rs1008949), dominant model, recessive model, and allele frequency showed no significant differences between patients with CAD and control participants (*P* > 0.05).

Table 5 shows the distribution of the genotypes and alleles for rs1996147 of the *Egr*3 gene in the Han and Uygur populations. Both in the CAD patients and in the control subjects, the distribution of genotype and alleles is significantly different in the two ethnic populations (all *P* < 0.001).

**Table 5:** Genotyping and allele distributions in the Han and Uygur population (rs1996147)

	**CAD patients**			**Control subjects**		
**Variants**	**Han population**	**Uygur population**	***P *** **value**	**Han population**	**Uygur population**	***P *** **value**
Genotyping						
AA	45(11.0%)	109(36.5%)		49(14.0%)	75(24.7%)	
AG	199(48.7%)	142(47.5%)		156(44.4%)	156(51.5%)	
GG	165(40.3%)	48(16.0%)	<0.001*	146(41.6%)	72(23.8%)	<0.001*
Allele						
A	289(35.3%)	360(60.2%)		254(36.2%)	306(50.5%)	
G	529(64.7%)	238(39.8%)	<0.001*	448(63.8%)	300(49.5%)	<0.001*

CAD, coronary artery disease; SNP, single-nucleotide polymorphism; *P < 0.05.

To further investigate the functional role of the rs1996147 genotypes associated with CAD in the Uygur, multivariable logistic regression analysis was performed (Table 6). In total subjects and males, following adjustments for the serum concentration of total cholesterol, LDL-C, and HDL-C, diabetes mellitus, essential hypertension, and smoking, the significance of the recessive model (AA vs GG + AG) between patients with CAD and control participants was retained (total: OR = 1.705, 95% CI: 1.166-2.494, *P* = 0.006; males: OR = 1.908, 95% CI: 1.189-3.062, *P* = 0.007).

**Table 6:** Multiple logistic regression analysis for CAD patients and control subjects in the Uygur population (rs1996147)

	**Total**			**Men**			**Women**		
**Risk factors**	**Odd ratios**	**95% CI**	***P *** **value**	**Odd ratios**	**95% CI**	***P *** **value**	**Odd ratios**	**95% CI**	***P *** **value**
Recessive model(AA vs GG + AG)	1.705	1.166-2.494	0.006*	1.908	1.189-3.062	0.007*	1.353	0.694-2.637	0.375
DM	1.363	0.85-2.184	0.199	1.442	0.787-2.64	0.236	1.288	0.589-2.816	0.525
Hypertension	1.209	0.849-1.723	0.293	1.205	0.772-1.88	0.413	1.206	0.666-2.187	0.536
TC	1.864	1.486-2.338	<0.001*	2.268	1.684-3.057	<0.001*	1.415	0.947-2.114	0.09*
LDL	1.046	0.856-1.278	0.662	1.017	0.806-1.282	0.887	1.122	0.697-1.805	0.636
HDL	0.413	0.222-0.766	0.005*	0.469	0.221-0.99	0.047*	0.354	0.117-1.067	0.065
Smoking	2.657	1.877-3.763	<0.001*	2.404	1.535-3.062	<0.001*	3.447	1.811-6.559	<0.001*

CAD, coronary artery disease; DM, diabetes mellitus; TC, total cholesterol; LDL, Low-density lipoprotein; HDL, high-density lipoprotein; *P < 0.05.

## Discussion

*Egr*3 is a member of the *Egr* family, whose members are rapidly induced by extracellular stimuli and have been implicated in the proliferation and differentiation of several different cell types, including endothelial cells. The *Egr*3 gene, located on chromosome 8p21.3, was cloned from a serum-activated cDNA library [21,22] and was originally described as a T-cell receptor–induced cyclosporine A-sensitive factor responsible for the upregulation of Fas ligand (FasL) [23]. Although *Egr*3 has been studied primarily in the context of lymphocyte and neuromuscular development, recent evidence points to a role for *Egr*3 in transducing signals in endothelial cells. For example, previous studies [24,25] have shown that *Egr*3 is one of the most highly inducible genes in VEGF–treated endothelial cells. Liu D et al. [7] reported that *Egr3*, most likely together with other Egr isoforms, plays an important role in angiogenesis. VEGF is essential for endothelial cell differentiation and angiogenesis during development and plays a major role in neovascularisation in a variety of disease states [26]. Lyn D et al. [12] reported that a cDNA mouse expression array containing 588 genes representing diverse biological functions was used to simultaneously compare changes in gene expression after 24 h of ischemia with heart tissue from sham-operated mice. The cDNA array experimental approach provided a global profile of gene expression changes in the heart ventricle tissue after coronary artery occlusion. Moreover, the expression of *Egr*1 and *Egr*3 was induced by ischemia. The mechanism by which *Egr*3 may be associated with CAD remains unclear.

In the present study, there was a significant difference in the genotypic distribution of rs1996147 between CAD patients and control subjects in the Uygur. We found that the *Egr3* polymorphisms (rs1996147) were associated with a risk of CAD in the Uygur population. For total participants and males in the Uygur group, the A allele frequency was higher in CAD patients than in control subjects. This implies that the risk of CAD is increased with the presence of the A allele in the Uygur population. Similarly, the distribution of the rs1996147 recessive model (AA vs GG + AG) was significantly higher in the patients with CAD than in the control subjects. The significant difference in the recessive model between the two groups still existed after the multivariate adjustment of confounding factors, such as plasma concentration of TC, HDL and LDL, incidence of hypertension, diabetes, and smoking. This result indicated that the risk of CAD may increase in Uygur males with the presence of AA at rs1996147. However, in our study, Uygur females did not display a difference in the allele frequency or genotypic distribution between CAD patients and control participants. This result may be related to the small sample size of Uygur females. However, the distribution of rs1996147 genotypes, dominant model, recessive model, and allele frequency showed no significant differences between the patients with CAD and the control participants in the Han group (P > 0.05).

The distribution of rs1008949 genotypes, dominant model, recessive model, and allele frequency did not show significant difference between the patients with CAD and the control subjects in the Han and Uygur groups. Our study is the first case–control study to investigate the association between the human *Egr*3 gene and CAD in the Uygur and Han in western China.

In conclusion, our study demonstrated that the rs1996147 polymorphism of the *Egr*3 gene was associated with CAD in males of the Chinese Uygur population. Males with the A allele might have a higher risk for CAD than those with the GG genotype. A larger sample size case–control study is required to investigate the relationship between the rs1996147 polymorphism of the *Egr*3 gene and CAD in females of the Uygur population. Additional studies need to be undertaken to clarify the underlying molecular mechanism that associates the *Egr*3 polymorphisms with CAD.

## Competing interests

The authors declare that they have no competing interests.

## Authors’ contributions

XL and XX carried out the molecular genetic studies and drafted the manuscript. YNY, XM and YYZ carried out the genotyping. SP and BDC participated in the design of the study and performed the statistical analysis. YTM, FL and XL conceived the study and participated in its design and coordination and helped to draft the manuscript. All authors read and approved the final manuscript.
